# Validation of the Turkish version of Endometriosis Health Profile Questionnaire (EHP-30) to evaluate the quality of life in women with endometriosis

**DOI:** 10.52054/FVVO.15.2.079

**Published:** 2023-06-30

**Authors:** E Darici, M.N.C. Kemahlı, P.Y. Bahat, B Yücel, E Oral

**Affiliations:** Centre for Reproductive Medicine Universitair Ziekenhuis Brussel, Vrije Universiteit Brussel, Belgium; Department of Obstetrics and Gynecology, University of Health Sciences, Istanbul Zeynep Kâmil Maternity and Childrens Hospital, Istanbul, Turkey; Department of Obstetrics and Gynecology, University of Health Sciences, Kanuni Sultan Süleyman Training and Research Hospital, Istanbul, Turkey; Department of Obstetrics and Gynecology, University of Health Sciences, Başakşehir Çam and Sakura City Hospital, Istanbul, Turkey; Department of Obstetrics and Gynecology, Bezmi-alem Vakif University, Istanbul, Turkey; Endometriosis & Adenomyosis Society, Turkey

**Keywords:** Endometriosis, questionnaire, translation, validity, quality of life

## Abstract

**Background:**

The Endometriosis Health Profile-30 (EHP-30) is a commonly used tool for assessing the impact of endometriosis on a person’s quality of life. The EHP-30 is a 30-item questionnaire that measures various aspects of endometriosis-related health, including physical symptoms, emotional well-being, and functional impairment.

**Objectives:**

EHP-30 has not yet been evaluated with Turkish patients. Therefore, we aim to develop and validate the Turkish version of EHP-30 in this study.

**Materials and Methods:**

This cross-sectional study was conducted with 281 randomly selected patients from Turkish Endometriosis Patient-Support Groups. The items of the EHP-30 distributed across 5 subscales of the core questionnaire are generally applicable to all women with endometriosis. There are 11 items on the pain scale, 6 on the control and powerlessness scale, 4 on the social support scale, 6 on the emotional well-being scale, and 3 on the self-image scale. The patients were asked to complete the form with brief demographic information and psychometric evaluation included factor analysis, convergent validity, internal consistency, test-retest reliability, data completeness and the determination of floor and ceiling effects.

**Main outcome measure:**

The main outcomes measures were the test-retest reliability, internal consistency, and the assessment of construct validity.

**Results:**

102 consecutive cases were analysed with no cases confirming intraluminal disease. Non-specific evidence of endometriosis such as tight angulation of the bowel was found in 36.3%. Following sigmoidoscopy 100 patients proceeded to surgery and the risk of bowel resection during surgery was 4%.

**Conclusion:**

In this study, 281 completed questionnaires were included with a return rate of 91%. Data completeness was accepted as excellent on all subscales. Floor effects were found in medical profession (37%), children (32%) and work (31%) modules. No ceiling effects were found. Division of the core questionnaire into five subscales identical to the original EHP-30 was confirmed by factor analysis performed. The intraclass correlation coefficient for agreement varied from 0.822 to 0.914. There was agreement between the EHP-30 and EQ-5D-3L on both of the hypotheses that were tested. There was a statistically significant difference in scores between endometriosis patients and healthy women across in all subscales (p<.01).

**What's new?:**

EHP-30 had not yet been evaluated with Turkish patients and the results of this study demonstrate the validity and reliability of the Turkish translation of the EHP-30 in assessing endometriosis patients’ health- related quality of life.

**ClinicalTrials Registration:**

NCT04862364.

## Introduction

Endometriosis is a long-term inflammatory disease characterized by the presence of endometrial tissue outside the uterine cavity. It affects approximately 5-10% of at reproductive period. Only 20-25% of endometriosis patients are asymptomatic, up to 80% suffer from chronic pain and 30-50% have infertility (Jia et al., 2012).

Endometriosis can have a significant impact on a person’s quality of life, causing physical discomfort and affecting their ability to work and engage in daily activities. In addition to the physical symptoms, endometriosis can also take a toll on a person’s mental health. Many women with endometriosis struggle with anxiety, depression and feelings of isolation due to the chronic nature of the condition and its impact on their daily lives. Despite the prevalence of endometriosis, it can take years for a diagnosis to be made, due in part to the lack of awareness about the condition and the difficulty in diagnosing it. This delay in diagnosis can compound the negative impact on a person’s quality of life as they struggle to manage their symptoms without a proper understanding of what is causing them. As a result, endometriosis is seen as a disabling disorder that can have a substantial impact on women’s daily lives, social interactions, sex life and mental health. Patients’ physical, mental and social elements of life affected by a medical condition or its treatment are all included in the multi-domain concept known as health-related quality of life (HRQoL) ) (Guidance for industry, 2006). There is currently no gold standard for assessing HRQoL in endometriosis patients (Jia et al., 2012). The identified tools for measuring HRQoL in endometriosis use a variety of distinct conceptual frameworks, scales, response formats, and scoring systems. This heterogeneity makes it difficult to adequately compare results and reach reliable conclusions. Within the context of endometriosis, their psychometric characteristics and internal consistency were not adequately established (Jia et al., 2012). Generic instruments which are useful for comparing different disorders have poor correlation with pain intensity, and being compromised by the use of medications in endometriosis limits their use, generic are not addressing highly-correlated issues associated with endometriosis such as infertility which constitutes a major limitation (Jones et al., 2001).

Endometriosis Health Profile-30 (EHP-30) was developed by Jones et al. (2001) to evaluate the health-related quality of life in women with endometriosis and it is the first standardised disease-specific instrument. The EHP-30 is a a 30- item questionnaire that measures various aspects of endometriosis-related health, including physical symptoms, emotional well-being, and functional impairment. The questionnaire is designed to capture the impact of endometriosis on a person’s life, providing a comprehensive picture of the effects of the condition on their health and well-being. The EHP-30 is a valuable tool for healthcare providers, as it allows them to track changes in a person’s symptoms over time and assess the effectiveness of treatment. It also provides individuals with endometriosis with a way to communicate their experiences to their healthcare provider, helping to ensure that they receive the care and support they need. European Society for Human Reproduction and Embryology (ESHRE) and the American Society for Reproductive Medicine (ASRM) have both endorsed using the EHP-30 in HRQoL studies on endometriosis due to the instrument’s high reliability, validity, and interpretability. (Jones et al., 2001; Vincent et al., 2010).

To the best of our knowledge, an endometriosis- specific, validated and a reliable instrument to measure HRQoL is not available in Turkey. Therefore, we aimed to develop and validate a Turkish version of the EHP-30 in this study.

## Materials and Methods

### The questionnaire

EHP-30 is a tool to measure different range of effects that endometriosis can have and consists of two parts: a core questionnaire and a modular questionnaire. The 30 items distributed across 5 subscales of the core questionnaire are generally applicable to all women with endometriosis. There are 11 items on the pain scale, 6 on the control and powerlessness scale, 4 on the social support scale, 6,on the emotional well-being scale and 3 on the self- image scale. There are also six extra models with an additional twenty-three items that may or may not be relevant to all women with endometriosis. Work module and sexual relationship addressed in 5 items whereas feelings about the medical profession addressed with 4, both feelings about treatment and feelings about infertility in 3 and the relationship with child/children addressed in 2. No score was assigned to a subscale if any of its items were left unanswered. The objective of the EHP is to assess the amount of self-reported illness. As a result, a conventional 0–100 scale is used, where 0 represents optimal health and 100 represents the worst health status.

### Translation and linguistic adaptation

After obtaining permission from the developer, Oxford University, the study was registered to the ClinicalTrials database with the identifier: NCT04862364. Translation process carried out in the following steps: First two professional independent translators translated the English version of EHP-30 into Turkish. The authors compared the translations of both translators. Minor discordance was detected, and authors then consolidated the two translations into one Turkish version. Then two translators performed back translation. After the backward translation a team consisting of all the authors and both translators reviewed the back translation and the latest form of the Turkish version of EHP-30 was released after the decision of semantic and conceptual equivalence of the terms. Pilot testing was conducted among 30 women between ages 18-45 who have histologically confirmed diagnosis of endometriosis by surgical intervention. All the interviews were face to face and carried out by one of the authors P.Y.B. At the final step the latest version EHP-30 was proofread and the original questionnaire remained unchanged.

### Ethics

The study received local ethical approval. (Kanuni Sultan Suleyman Hospital ethical committee: KAEK/2021.04.149). All women were informed of the study’s objectives and informed that their participation was entirely voluntary, and they were allowed to withdraw at any period. The patients were given written instructions to ensure they understood the process and to reassureance about the confidentiality.

### Study design and data collection

This cross-sectional study was carried out at Kanuni Sultan Suleyman Research and Training Hospital between January 2020 to January 2021. All women included in the study were randomly selected from Turkish Endometriosis Patient-Support Groups. The patients were asked to complete a form with brief demographic information on their age, marital and reproductive status and family history as well as both the EHP-30 and EQ-5D-3L questionnaires.

### Psychometric evaluation

- Sample Size: Estimating that 10% of reproductive-aged women have endometriosis allowed for a sufficient sample size to be determined. OpenSource Epidemiologic Statistics for Public Health (OpenEpi) software was used to determine the sample size. To achieve 80% power and a 95% confidence interval, a sample size of 242 patients is required for the study.
- Factor Analysis: To analyse the factor structure of the Turkish EHP-30 subscales, principal component factor analysis with varimax rotation was used. Those items with loadings greater than 0.40 were given.
- Convergent validity: The assessment of construct validity was performed using the European Quality of Life-5 Dimensions -3 questionare (EQ- 5D-3L), which is a widely recognized health-related quality of life (HRQoL) tool that has undergone extensive validation for the Turkish language. It was hypothesized that there would be a significant correlation between the EHP-30 and EQ-5D-3L with respect to the following five sub hypotheses the EHP-30 and EQ-5D-3L with respect to the following five sub hypotheses: EHP-30 subscale pain with EQ-5D-3L subscales pain/discomfort and daily activities; EQ-5D-3L subscale anxiety/depression; EHP-30 Control. The correlation between the two groups was determined using Spearman’s rho.
- Internal consistency: Each scale’s internal consistency was calculated using Cronbach’s alpha, and scores above 0.70 indicated adequate internal consistency (Cronbach and Warrington, 1951).
- Test-retest reliability: Test-retest reliability was evaluated by calculating the intraclass correlation coefficient (ICC) for agreement between two questionnaires filled out by the same set of endometriosis patients 1 month apart. Considering that endometriosis complaints may be related to a woman’s menstrual cycle, a 30-day window was selected. The Wilcoxon signed rank test was used to determine the statistical significance of differences in scores between at time points 1 and 2.
- Descriptive statistics: Data are presented in Mean ± SD. If applicable, data were compared with a t-test or a nonparametric test (Mann-Whitney test) in case of skewed data. All statistical analyses were performed with SPSS 22 for Mac-Os (SPSS., Chicago, IL). P values of <.05 were considered statistically significant.

## Results

### Participants

The study included 281 completed questionnaires and all participants were Turkish. The patient return rate was 91% and 80 women without any health complaints were selected from those who visited the hospital for routine check-ups. Both the patient group and the healthy women group had comparable age and marital status. Women who were healthy were more likely to be paid employees or volunteers.

### Data completeness, score distributions and floor and ceiling effects

An excellent data completeness was attained across all subscales. Only 1.7% of responses were absent on the subscales of the modular questionnaire, whereas 0.6% were missing from the core questionnaire. Sixty-one percent of the questions were fulfilled by all the responders. Three out of eight modules in the modular questionnaire revealed floor effects: medical profession (37%), children (32%), and work (31%). There was no ceiling effect.

Table I shows the distribution of scores on the Turkish version of the Endometriosis Health Profile-30 for women with and without the disease. In most subscales, the distribution of scores encompassed’s the entire possible range (0-100).

**Table I:** Score distribution on the Turkish version of Endometriosis Health Profile–30 of endometriosis patients and healthy women.

	Subscales	Endometriosis Patients (n=281)	Healthy Women (n=80)	P value**
Core Questionnaire	Pain (Q1-11)	29 (17-39)	0 (0-1)	<0.001
	Control and powerlessness (Q12-17)	16 (9-20)	0 (0-0)	<0.001
	Emotional well-being (Q18-21)	16 (11-21)	1 (0-2)	<0.001
	Social support (Q22-27)	11 (7-14)	0 (0-0)	<0.001
	Self-image (Q28-30)	7 (5-11)	0 (0-1)	<0.001
Modular Questionnaire	Work (Q1-5)	6 (1-13)	0 (0-1)	<0.001
	Children (Q6-7)	3 (0-6)	0 (0-1)	<0.001
	Sexual intercourse (Q8-12)	7 (0-13)	0 (0-1)	<0.001
	Medical profession (Q13-16)	5 (0-11)	0 (0-1)	<0.001
	Treatment (Q17-20)	4 (0-8)	0 (0-1)	<0.001
	Infertility (Q20-23)	3 (0-11)	0 (0-1)	<0.001

*Values are given as median(range), **Mann-Whitney U Test.

### Factor analysis

The core questionnaire was divided into five subscales that were identical to those in the original EHP-30, according to factor analysis. The original EHP-30 classified the question about “feeling violent or aggressive” under the emotion subscale, however the present data suggests that it more properly belongs in the social support subscale. Cronbach’s coefficient for the subscales ranged from 0.75 to 0.97. Coefficients were above 0.90 in eight of the eleven subscales.

### Convergent validity

The results of the comparison between the EHP- 30 and the EQ-5D-3L supported both of the tested hypotheses, with a statistically significant difference in scores observed between endometriosis patients and healthy women in all of the EHP-30 subscales (P<.01). The correlations between the EHP-30 and the EQ-5D-3L, as measured by the Spearman correlation coefficients, ranged from 0.44 to 0.63 for all nine sub-hypotheses (P<.01). The highest correlations were seen between similar subscales such as the EHP-30’s pain and the EQ-5D-3L’s pain/ discomfort subscales (Table II).

**Table II:** Assessment of construct validity Turkish version of the Endometriosis Health Profile–30 (n=281) with Turkish version of The European Quality of Life–5 Dimensions -3 Levels questionnaire (EQ-5D-3L) (n=281).

Compared subscales		Spearman’s rho correlation coefficient value	P value
EHP-30 Pain	EQ-5D-3L Pain/Discomfort	0.384	<0.001
EHP-30 Pain	EQ-5D-3L Daily Activities	0.395	<0.001
EHP-30 Control And Powerlessness	EQ-5D-3L Anxiety/Depression	0.284	<0.001
EHP-30 Emotional Well-Being	EQ-5D-3L Anxiety/Depression	0.341	<0.001
EHP-30 Social Support	EQ-5D-3L Anxiety/Depression	0.361	<0.001
EHP-30 Self-Image	EQ-5D-3L Anxiety/Depression	0.324	<0.001

### Internal consistency and test-retest reliability

47 out of 55 questionnaires (87%) were reperformed to determine test-retest reliability (Table III). The ICC for agreement varied from 0.822 to 0.914 (Table III). Scores on five of the subscales were significantly different between time points 1 and 2 according to the Wilcoxon signed rank test (Table III). In most subscales, scores were consistently lower during the second measurement. The difference, however, did not reach 5% of the entire score range and is unlikely to reflect a clinically relevant difference.

**Table III:** Test-retest reliability, internal consistency, and statistical significance of differences between scores of test-retest with Wilcoxon signed rank test for Turkish version of the Endometriosis Health Profile–30 (EHP-30) (n=281).

Sub-scales	Pain (Q1-11)	Control and powerlessness (Q12-17)	Social support (Q18-21)	Emotional well-being (Q22-27)	Self-image (Q28-30)
Intraclass correlation coefficients	0.995	0.998	1	1	0.999
Cronbach’s alpha Values	0.885	0.822	0.84	0.914	0.853
Wilcoxon signed rank test P value	0.160	0.180	1	1	0.317

The analysis of the correlation between patients’ subscale pain score for Turkish version of EHP–30 and the pain Visual Analog Scale have showed no statistically significant differences (Table IV).

**Table IV:** Correlation between patients’ subscale pain score for Turkish version of the Endometriosis Health Profile–30 and The Pain Visual Analog Scale (VAS).

Subscale pain score for Turkish version of Endometriosis Health Profile–30	The Pain Visual Analog Scale	Spearman’s rho correlation coefficient value
n=281	n=281	< 0.01

## Discussion

As one of the well accepted questionnaires used to measure the specific impact of endometriosis on HRQoL, the EHP-30 has been translated into many languages including Chinese, Brazilian, Dutch, Portuguese, Swedish, French and Spanish (Mengarda et al., 2008; van de Burgt et al., 2011; Jia et al., 2012; Nogueira-Silva et al., 2015; Chauvet et al., 2017; Grundström et al., 2020; Marí-Alexandre et al., 2022). When compared with other scales Jones et al. (2001) suggested that the EHP-30 has a high degree of internal consistency and test–retest reliability and provide a more comprehensive and rigorous assessment of HRQoL, which is crucial in clinical research (Jones et al., 2001). In a recent systematic review Bourdel et al. (2019) indicated that the EHP- 30 is not only the most commonly used questionnaire but also the most validated questionnaire for measuring HRQoL among endometriosis patients. For the above-mentioned reasons, we decided to translate and validate the EHP-30 into Turkish. Eight quality criteria are outlined in the recommendations for determining the validity of an HRQoL questionnaire. As suggested by Jones et al. (2001) and implied to similar studies previously, five criteria were used to assess the psychometric properties of the Turkish version of the EHP-30 questionnaire, namely factor analysis, convergent validity, internal consistency, test-retest variability, and score distributions (floor and ceiling effects). Due to the absence of a gold standard for evaluating the HRQoL in women with endometriosis, a criterion-validity evaluation of the EHP-30 could not be performed. In our study the return rate of %91 was achieved. High response rates and data completeness indicated that the questionnaire was easy to comprehend and accept. No evidence of floor and ceiling effects were present, which suggests that changes in the health status of women with the lowest level can be accurately measured, although changes in the health status of women with the highest level may not be easily noticeable. As established previously in Dutch, French, Chinese, Portuguese and Persian versions (Nojomi et al., 2011; van de Burgt et al., 2011; Jia et al., 2013; Nogueira-Silva et al., 2015; Chauvet et al., 2017) the factor analysis in our study confirmed the division of the core questionnaire into five subscales whereas a four-factor model was confirmed for the Swedish version (Grundström et al., 2020) and a three-factor model for the Norwegian (Cronbach and Warrington, 1951) version. The coefficient was 0.90 or higher in eight of the eleven subscales, suggesting that these subscales may be appropriate for individual-level analysis.

Our data showed good internal consistency. Test- retest reliability was high (0.822 to 0.914) and the lowest ICC value of the present study was higher when compared with the previously conducted studies (van de Burgt et al., 2013; Chauvet et al., 2017; Verket et al., 2018) showing acceptable to excellent test-retest reliability. Scores were systematically lower during the second measurement in most subscales; however, this was not likely to represent a clinically-relevant difference (Koo and Li, 2016)

One of the limitations of our study was the method of recruitment. In our study, the participants were primarily sourced from the Turkish Endometriosis Patient-Support Group which may lead to a selection bias because women with severe symptoms are more likely to seek help. Another point to consider is any symptom that is attributable to endometriosis may result from another unconfirmed comorbidity since thorough medical evaluation could not be performed.

## Conclusions

The results of this validation study for the EHP- 30 indicated a high level of data completeness, with no significant floor or ceiling effects. The questionnaire demonstrated good internal consistency and excellent test-retest reliability. These findings confirm that the Turkish version of the EHP-30 is a valid and reliable tool for measuring the health-related quality of life in individuals with endometriosis.
